# Identification of high-risk cells in single-cell spatially resolved transcriptomics data using Diagnostic Evidence GAuge of Single-cells with spatial smoothing

**DOI:** 10.1093/bioinformatics/btag098

**Published:** 2026-04-03

**Authors:** Debolina Chatterjee, Justin L Couetil, Ziyu Liu, Kun Huang, Chao Chen, Jie Zhang, Michael A Kalwat, Travis S Johnson

**Affiliations:** Department of Biostatistics and Health Data Science, Indiana University School of Medicine, Indianapolis, IN, 46202, United States; Department of Medical and Molecular Genetics, Indiana University School of Medicine, Indianapolis, IN, 46202, United States; Indiana Clinical and Translational Sciences Institute, Indianapolis, IN, 46202, United States; Department of Statistics, Purdue University, West Lafayette, IN, 47907, United States; Department of Biostatistics and Health Data Science, Indiana University School of Medicine, Indianapolis, IN, 46202, United States; Department of Medical and Molecular Genetics, Indiana University School of Medicine, Indianapolis, IN, 46202, United States; Melvin and Bren Simon Comprehensive Cancer Center, Indianapolis, IN, 46202, United States; Center for Computational Biology and Bioinformatics, Indiana University School of Medicine, Indianapolis, IN, 46202, United States; Department of Biomedical Informatics, Stony Brook University, Stony Brook, NY, 11794, United States; Department of Medical and Molecular Genetics, Indiana University School of Medicine, Indianapolis, IN, 46202, United States; Melvin and Bren Simon Comprehensive Cancer Center, Indianapolis, IN, 46202, United States; Center for Computational Biology and Bioinformatics, Indiana University School of Medicine, Indianapolis, IN, 46202, United States; Indiana Biosciences Research Institute, Indianapolis, IN, 46202, United States; Center for Diabetes and Metabolic Diseases, Indiana University School of Medicine, Indianapolis, IN, 46202, United States; Department of Biochemistry and Molecular Biology, Indiana University School of Medicine, Indianapolis, IN, 46202, United States; Department of Biostatistics and Health Data Science, Indiana University School of Medicine, Indianapolis, IN, 46202, United States; Melvin and Bren Simon Comprehensive Cancer Center, Indianapolis, IN, 46202, United States; Center for Computational Biology and Bioinformatics, Indiana University School of Medicine, Indianapolis, IN, 46202, United States; Indiana Biosciences Research Institute, Indianapolis, IN, 46202, United States

## Abstract

**Summary:**

The examination of high-risk cells and regions in tissue samples from spatially resolved transcriptomics platforms offers meaningful insights into specific disease processes. For existing methods, while cell types or clusters can be identified and associated with disease attributes, individual cells are unable to be associated in the same manner.

**Method:**

Diagnostic Evidence Gauge of Single-Cells and Spatial Transcriptomics (DEGAS) solves the above problem by employing latent representations of gene expression data and domain adaptation to transfer disease attributes from patients to individual cells from single-cell RNA sequencing datasets. In this research, we present and evaluate DEGAS’s versatility in adapting to data arising from various single-cell spatially resolved transcriptomics (scSRT) platforms. DEGAS successfully identified high-risk cells and regions in liver hepatocellular carcinoma and skin cutaneous melanoma, which were validated through known markers. Additionally, DEGAS was applied to our newly generated Type II Diabetes Xenium dataset, revealing high-risk cells within the tissue samples.

**Availability and implementation:**

The DEGAS software can be accessed at https://github.com/tsteelejohnson91/DEGAS. For the updated smoothing functions and associated codes, visit https://github.com/dchatter04/DEGAS-Spatial-Smoothing, which is archived at https://doi.org/10.5281/zenodo.18510221. Sources for the datasets reviewed are detailed in their respective sections. A description of some datasets, along with extra tables and figures, is provided in the Supplementary Materials file. Our newly generated Xenium data for Type II Diabetes can be found at https://doi.org/10.7303/syn68699752.

## 1 Introduction

Single-cell spatially resolved transcriptomics (scSRT) merges sequencing and imaging techniques to study tissue samples with high precision, offering crucial insights into disease mechanisms. Over recent years, scSRT data have evolved to include both spatial and gene expression details, along with related histological images of tissue specimens. Popular platforms that provide such datasets include 10X Genomics’ Xenium (Janesick et al. 2023), MERFISH (Chen et al. 2015), segFISH+ (Eng et al. 2019), and Nanostring Technologies’ CosMx (He et al. 2022), among others. Although these data give detailed information about single cells, patient-level attributes such as overall survival or other physiological characteristics cannot be directly attributed to individual cells. Furthermore, these datasets are limited by sample size, making it difficult to generalize findings. On the other hand, bulk RNA-seq datasets from platforms like the Gene Expression Omnibus (GEO) or The Cancer Genome Atlas (TCGA) reveal information on diverse disease characteristics for patients, such as diagnostic details like disease subtype and status, and prognostic information like survival and treatment responses, etc., but they lack granular single-cell details. This limitation hinders the identification of cell subsets linked to disease characteristics, especially when disease-related cells are mixed with non-disease-related cells. Thus, there is a pressing need for methods to translate information from scSRT data to the patient level. Using the rationale that single-cell RNA-seq (scRNA-seq) data and patient-level transcriptomic data (such as bulk RNA-seq with clinical annotations) share the same gene set and, therefore, a common feature space, there is a natural link allowing efficient information transfer between these data types to uncover associations between patients and cells. Diagnostic Evidence Gauge of Single-Cells and Spatial Transcriptomics (DEGAS) (Johnson et al. 2022) uses transfer learning methods like domain adaptation (Kouw and Loog 2021) and multitask learning (Caruana 1997) to link individual cells to disease risk for scRNA-seq data. In this article, we demonstrate how DEGAS was modified to utilize the spatial information from scSRT datasets and its utility in liver hepatocellular carcinoma (LIHC), skin cutaneous melanoma (SKCM), and Type II Diabetes (T2D). The sources of the respective datasets are revealed in the subsequent sections.

## 2 Materials and methods

**Figure 1:**
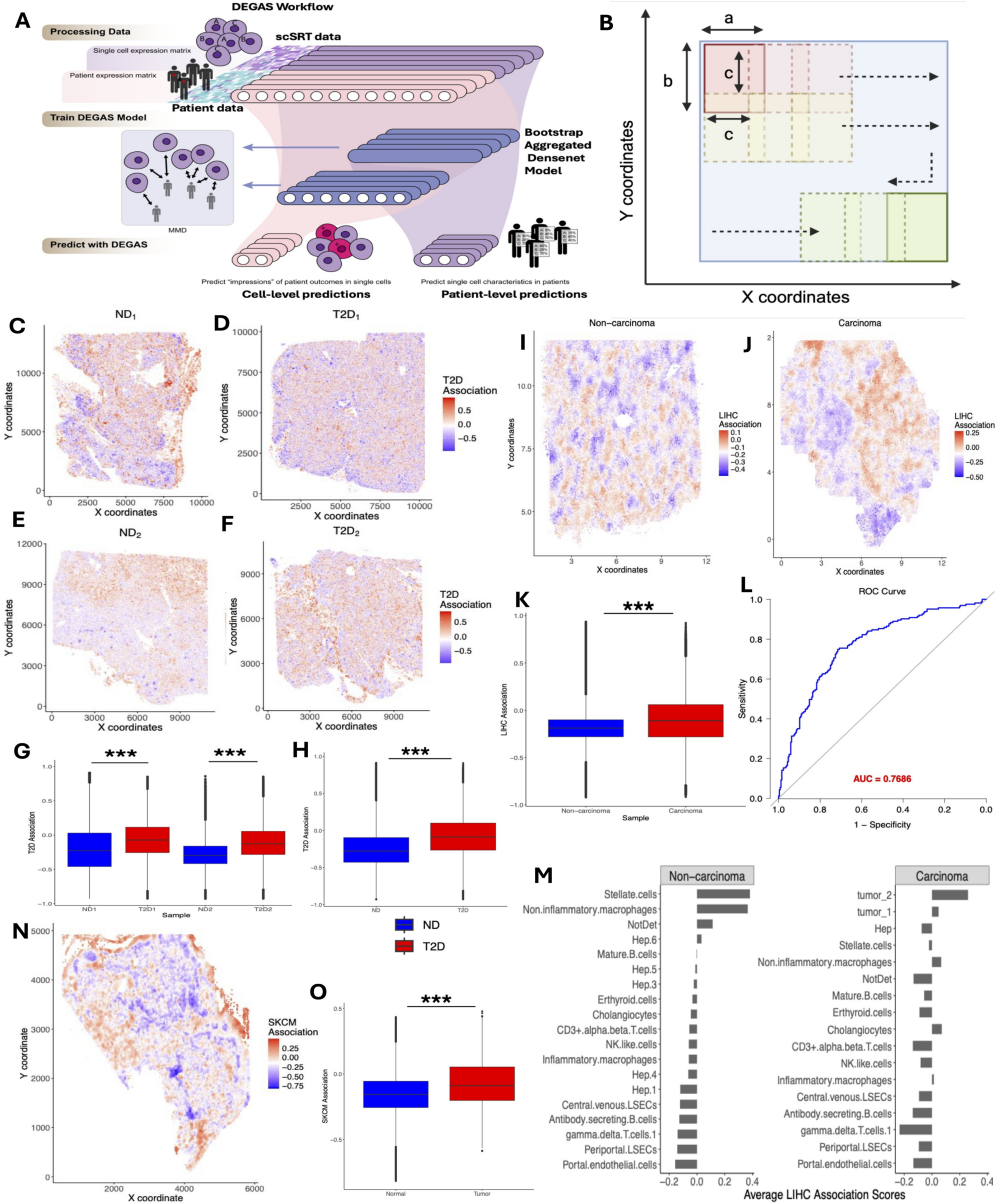
(A) The DEGAS workflow, showing the key inputs and outputs, is overall similar to that of Johnson et al. (2022). (B) Sliding windows progressively shift to capture the entire tissue, enabling localized spatial smoothing of the disease association scores for SWiS and FoVs algorithms. (C–F) Spatial visualization of T2D association scores generated by the DEGAS+SWiS model for Xenium tissue samples: $ND_{1}$ (panel C), $T2D_{1}$ (D), $ND_{2}$ (E), and $T2D_{2}$ (F), respectively. Legends display the scaled T2D disease association scores. (G) Boxplot showing a significant difference in T2D association scores across samples, with higher scores observed in T2D samples compared to non-diabetic samples (*** indicates significant *P*-values). (H) Combining disease association scores from non-diabetic and T2D samples separately reveals higher overall T2D association scores in diabetic samples. (I and J) Spatial visualization of LIHC association scores generated by the DEGAS+FoVS model for CosMx tissue samples: normal (panel I) and LIHC carcinoma (panel J), respectively. (K) LIHC association scores for the carcinoma sample are significantly higher than those of the non-carcinoma sample. (L) ROC curve showing patient-level survival prediction by the DEGAS model for classifying 3-year overall survival status in Liver CosMx data. (M) LIHC association scores by various cell types across the non-carcinoma and carcinoma samples, respectively. (N) Spatial visualization of SKCM association scores generated by the DEGAS+SWiS model for a Xenium tissue sample. (O) The SKCM association scores for cells within the tumor region are significantly higher than those in the stromal and other non-tumor regions.

The DEGAS framework (Fig. 1A) is designed using Python’s TensorFlow library and provides an interface in R. In this article, we skip the detailed explanation of the framework, as it can be found in the original publication, Johnson et al. (2022).

The proposed DEGAS+smoothing framework comprises two main stages. In the first stage, the DEGAS model is trained on single-cell spatially resolved transcriptomics (scSRT) data, using only gene expression information to generate disease association scores for each individual cell. The DEGAS model preprocessing and training pipeline is largely the same between scRNA-seq and scSRT data (see Algorithm 1, available as supplementary data at *Bioinformatics* online). The shared gene set between patient bulk RNA-seq data and scSRT data are chosen by either choosing the common high variance genes among the two datasets or common differentially expressed genes across disease conditions. After training the DEGAS model for each single cell, the patient-level disease association (DA) scores are predicted. Higher DA scores are interpreted as higher risk of the disease. In the second stage, we perform post-processing to include spatial information from scSRT technologies to account for spatial heterogeneity in the tissue, as adjacent cells with similar biological contexts may exhibit abrupt differences in their predicted scores due to technical noise and local variability in transcript capture. The key modification required to adapt to spatial datasets lies in the post-processing steps.

### 2.1 Postprocessing

We denote the DA scores, which are scaled within [−1,1] by $S^{\text{scaled}}$. We define windows based on pixel coordinates from the metadata and locally smooth the DA scores by the Spatial Window Smoothing algorithm below:

#### 2.1.1 SWiS algorithm

Let $C=\{c_{1},c_{2},\ldots ,c_{N}\}$ denote the collection of *N* cells. Each cell $c_{i}$ is associated with spatial coordinates $(x_{i},y_{i})\in R^{2}$, and the DA acores $S_{i}^{\text{scaled}}\in R$, where R is the set of real numbers. Let D be set of all cells, where each cell is represented by its spatial coordinates and DA scores, i.e. $D={\left\{(x_{i},y_{i},S_{i}^{\text{scaled}})\right\}}_{i=1}^{N}$, and focus on a small patch of the tissue that spans a 2-dimensional window with $x_{\min}=\underset{i}{\min}x_{i},\;x_{\max}=\underset{i}{\max}x_{i},\;y_{\min}=\underset{i}{\min}y_{i},\;y_{\max}=\underset{i}{\max}y_{i}$. A window starting at position (x,y) defines a rectangular patch $W_{x,y}=\left\{(x_{i},y_{i},S_{i}^{\text{scaled}})\in D|x<x_{i}<x+a,\;y<y_{i}<y+b\right\}$, where *a* is window width (X-axis range), *b*: window height (Y-axis range), *c* is step size (stride along X and Y) (as displayed in Fig. 1B). To ensure the sliding window works well with adequate number of cells (at least $n_{\min}$), while defining the window, we exclude cells that are too close to the boundary of the window, we use an internal padding *p* to only consider the cells that are in the *core* of it. The *core* of the window, excluding marginal cells is defined as $W_{x,y}^{\text{core}}=\{(x_{i},y_{i},S_{i}^{\text{scaled}})\in W_{x,y}|x+p<x_{i}<x+a-p$, $\;y+p<y_{i}<y+b-p\}$.

Starting at the top-left, the window moves rightward until it covers the X-range, then steps down by *c* along the Y-axis, repeating the process until all cells are included. Therefore, the horizontal shift is x→x+c until $x+a>x_{\max}$, and vertical shift is y→y+c, resetting $x=x_{\min}$. This generates a collection of windows ${\left\{W_{j}\right\}}_{j=1}^{J}$, each with a core region $W_{j}^{\text{core}}$.

Within each window $W_{j}^{\text{core}}$, define the set of core cells $C_{j}=\left\{i|(x_{i},y_{i})\in W_{j}^{\text{core}}\right\}$. The Algorithm 1 provides a step-by-step description of the SWiS algorithm.

Algorithm 1 SWiS: Spatial Window Smoothing

**Inputs:** 

- Cell data $C={\left\{(x_{i},y_{i},S_{i}^{\text{scaled}})\right\}}_{i=1}^{N}$
- Parameters: window width *a*, height *b*, stride *c*, padding *p*
- Thresholds: $n_{\min}$ (min cells per core), *k* (nearest neighbors)

**Initialize:** For each cell *i*, initialize list of smoothed scores $S_{i}\leftarrow \emptyset$ (where ∅ is the empty set)

**Sliding Window Loop:** 

**for** The $j^{th}$ window j=1,2,…,J with step size *c* **do** 

 Define window $W_{j}$: cells with $x<x_{i}<x+a$, $y<y_{i}<y+b$

 Define core $W_{x,y}^{\mathrm{core}}$: cells with $x+p<x_{i}<x+a-p$, $y+p<y_{i}<y+b-p$

 **if** number of cells in $W_{j}^{\text{core}}<n_{\min}$  **then** 

  Skip this window

 **end if** 

 **for** each core cell $c_{i}$ in $W_{x,y}^{\mathrm{core}}$  **do** 

  Identify *k* nearest neighbors of cell $c_{i}$ in the $j^{th}$ window as $N_{k}^{k}(c_{i})$ within $W_{j}^{\text{core}}$ (excluding $c_{i}$)

  Compute local smoothed value:

$${\tilde{S}}_{i}^{\left(j\right)}=\frac{1}{k}\sum_{c_{l}\in N_{j}^{k}(c_{i})}S_{l}^{\text{scaled}}$$

  Append ${\tilde{S}}_{i}^{\left(j\right)}$ to $S_{i}$

 **end for** 

**end for** 

**Final Averaging:** 

**for** each cell $c_{i}$  **do** 

 **if**  $S_{i}\neq \emptyset$  **then** 

  Compute average over all smoothed estimates:

$${\tilde{S}}_{i}=\frac{1}{\left|S_{i}\right|}\sum_{{\tilde{S}}_{i}^{\left(j\right)}\in S_{i}}{\tilde{S}}_{i}^{\left(j\right)}$$

 **else** 

  Set ${\tilde{S}}_{i}=S_{i}^{\text{scaled}}$  ▹ If cell was not included in any core

 **end if** 

**end for** 

**Output:** Final smoothed values ${\tilde{S}}_{i}$ for all i=1,2,…,N

Cells not included in any window can either be excluded from the final output or only have their raw scores be considered. A cell can have a list of smoothed DA scores due to overlapping windows, and these are averaged over all overlapping windows. This ensures full slide coverage while preserving local expression through spatially aware smoothing.

**FoVS Algorithm:** For datasets that are naturally divided into Fields of View (FOVs), instead of defining the sliding window, we use the naturally defined FOVs, while other computations remain the same as in the SWiS Algorithm 1. Each FOV has its own spatial coordinates and is treated as a discrete region. After smoothing within individual FOVs, the results are combined to reconstruct the full tissue. Next, we present the applicability of DEGAS+SWiS/FoVS in identifying high-risk cells across various diseases and data types.

## 3 DEGAS identifies high-risk pancreatic cells associated with type II diabetes using analysis of Xenium data

DEGAS preprocessing steps were conducted on the newly generated Xenium T2D data (described in Section 2, available as supplementary data at *Bioinformatics* online), including filtering for high-quality cells and normalizing gene expression to account for technical biases and ensuring robust downstream analysis. For the patient-level bulk RNA-seq data, clinical metadata obtained from the GEO dataset GSE159984 (Marselli et al. 2020), which includes data on 58 non-diabetic (ND) and 27 Type II Diabetic (T2D), was used. Clinical information for this bulk RNA-seq data included body mass index (BMI), sex, and T2D status. The shared gene set was identified through common highly variable genes in the bulk RNA-seq and scSRT datasets.

There were no cell-type labels in the scSRT data, and the bulk RNA-seq data included T2D status classes (T2D versus ND); we therefore employed the 5x BAg 3-layer *BlankClass* DEGAS model. This model predicted the likelihood of each individual cell being categorized as associated with T2D or ND. We applied SWiS on the DEGAS-predicted T2D association scores, with width and height 1000, stride 500, padding 100, 50 minimum cells, and 5 nearest neighbors.

The spatial plot of the DEGAS T2D association scores highlighted individual cells as low T2D-association (blue) or high T2D-association (red) (Fig. 1C–F). The Wilcoxon rank-sum test revealed that the average T2D-association scores for T2D samples were significantly higher than those of the ND samples (*P* < .001, Fig. 1G and H). This shows the viability of the DEGAS+SWiS approach.

We define low-risk cells as those with predicted T2D association scores below the 25th percentile and high-risk cells as those with scores above the 75th percentile. We chose these quartile-based thresholds instead of using the median because they provided the best trade-off between larger log2 fold changes (Log2FC) and a sufficient number of differentially expressed genes (DEGs) between the high- versus low-risk cells (see Table 2, available as supplementary data at *Bioinformatics* online). For the differential expression analysis, we only include genes expressed in at least 10% of cells.

Tables 2 and 3, available as supplementary data at *Bioinformatics* online, list genes that are significantly upregulated or downregulated (i.e. Benjamini–Hochberg adjusted *P*-value ≤ .05) in the high-risk regions of the Xenium samples. These tables also report the log_2_-fold changes in expression of these genes between high-risk, low-risk, and remaining cells. Figures 1–4, available as supplementary data at *Bioinformatics* online, present the corresponding volcano plots. Several of the genes that are significantly up- or downregulated in high-risk cells have previously been associated with T2D, including *PTPRC*  NCBI (2024), *AMY2A* (Yadav et al. 2013, Abdulkareem et al. 2024), and *CFTR* (Ntimbane et al. 2009, Gu et al. 2021). Observing these genes among the high-risk cells identified by our algorithm provides additional support for its effectiveness in detecting T2D risk-associated cells.

## 4 DEGAS identifies high-risk cell types in CosMx hepatocellular carcinoma samples

CosMx data are characterized by multiple FOVs that collectively cover the entire tissue area. Within each FOV, individual cells are mapped with precise FOV-specific spatial coordinates and the locations of individual RNA molecules within those cells. We obtained this publicly available LIHC data (He et al. 2022) (see Section 3, available as supplementary data at *Bioinformatics* online for details). The bulk RNA-seq data with clinical metadata were acquired from the (TCGA LIHC study). This dataset included data from 377 human individuals, along with their clinical information, including sex, age, overall survival status, and time. The shared gene set was identified through common highly variable genes in the bulk RNA-seq and scSRT datasets.

The gene expression count data for both samples was individually preprocessed according to DEGAS’s specified preprocessing steps mentioned in Algorithm 1, available as supplementary data at *Bioinformatics* online. However, there were no labels distinguishing individual single cells as tumor or normal. Thus, following the identification of a common highly variable gene set between the scSRT and the bulk RNA-seq data, the 5x BAg 3-layer *BlankCox* model of DEGAS was utilized. For CosMx data, due to the presence of well-defined FOVs and FOV-specific pixel coordinates, we used the naturally defined FOVs to smooth the LIHC-associations, with 50 nearest neighbors. The spatial map of DEGAS+FoVS LIHC-association scores (Fig. 1I and J) reveals high LIHC-association regions (red) and low LIHC-association regions (blue) for all cells within a field of view in both the non-carcinoma and LIHC samples. As expected, there is a highly significant difference in the LIHC-association scores between the non-carcinoma and LIHC samples (*P* < .001, Fig. 1K). Like Section 3, we use the 25th/75th percentile cutoff for the LIHC-association scores to separate low- and high-risk cells respectively. In the non-LIHC sample, 83 219 low- and 83 219 high-LIHC-association cells were identified, while in the LIHC sample, 115 750 low LIHC-association cells and 344 691 high LIHC-association cells were identified. This result reveals a significantly higher number of high LIHC-association cells in the cancer sample ($\chi^{2}=34873.64$, *P* < .001) compared to the non-carcinoma sample. For patient-level T2D association predictions using the DEGAS model, we utilized binary 3-year survival information from the TCGA dataset. Overall survival time was used as the ground truth, and we excluded censored patients to ensure reliable labeling. Patients who survived beyond 3 years were labeled as 0, while those who experienced an event (death) within 3 years were labeled as 1. The Receiver operating characteristic-area under the curve (ROC-AUC) was calculated considering the predicted DEGAS patient-level LIHC-association scores. The DEGAS model demonstrated strong discriminatory performance, achieving a high ROC-AUC of 0.7686 (Fig. 1L).

This publicly available dataset was provided with individual cell-type annotations and images (see Figs 5 and 6, available as supplementary data at *Bioinformatics* online) with their overlay in the tissue. The mean LIHC-association scores for each cell type within the liver tissues were calculated (Fig. 1M). Stellate cells and macrophages, representing stromal and immune cells, exhibited elevated scores in the non-carcinoma sample. In the LIHC (carcinoma) sample, tumor cells, non-inflammatory macrophages, and cholangiocytes showed higher average scores compared to endothelial or gamma delta T cells (Fig. 1M). High LIHC-associated spatial regions predicted by DEGAS in the carcinoma sample (Fig. 1J) overlapped with tumor cell–rich areas annotated by NanoString (see Fig. 6, available as supplementary data at *Bioinformatics* online). Similarly, in non-carcinoma sample regions exhibiting higher average LIHC association scores (Fig. 1I) were enriched with stellate cells, macrophages, mature B cells, and erythroid cells (see Fig. 5, available as supplementary data at *Bioinformatics* online). Macrophages, in particular, are known to contribute to tumor growth by suppressing immune surveillance through the inhibition of cytotoxic T cells (Ugel et al. 2015). A similar pattern was observed in a DEGAS-based study of prostate cancer, where specific normal tissues were linked to cancer progression (Couetil et al. 2023). In that study, normal regions with high disease-association scores were found to be infiltrated by macrophages, detectable in both transcriptomic data and histopathological images (Couetil et al. 2023). In our study, we observed a comparable trend. Furthermore, we applied the copyKAT algorithm (Gao et al. 2021) to distinguish aneuploid from diploid cells (see Section 1.3.1 and Fig. 7, available as supplementary data at *Bioinformatics* online). Although the analysis was limited by the availability of only ∼1000 genes, which reduced the confidence of copyKAT predictions, comparison of DEGAS+FoVs–predicted LIHC association scores between aneuploid and diploid cells revealed a statistically significant difference (*P* = .0348; see Fig. 8, available as supplementary data at *Bioinformatics* online). Despite the limited resolution of the copyKAT analysis due to the restricted number of detected genes, the observation that DEGAS+FoVs predicted significantly higher LIHC association scores in aneuploid cells supports the robustness of our framework. This finding indicates that DEGAS can effectively capture tumor-associated transcriptional and spatial patterns, even in datasets with reduced gene coverage.

## 5 DEGAS identifies high-risk cells in skin cutaneous melanoma samples

For this analysis, the bulk human SKCM data were acquired from TCGA SKCM study. This reference data contained 293 patients and their overall survival in months. The Xenium scSRT data was sourced from a publicly available 10X Xenium FFPE Human Skin dataset (https://www.10xgenomics.com/) which consists of a single tissue sample. There were no known cell labels for scSRT data, and bulk RNA-seq data has survival times; therefore, we applied the *BlankCox* DEGAS model, with overall survival as the patient-level outcome. The SKCM-association scores were calculated, and the SWiS algorithm was applied to find the smoothed DEGAS hazard scores. The SWiS SKCM-association scores were overlaid with the tissue’s spatial coordinates, highlighting areas of higher SKCM association within the tissue (Fig.1N). Our results demonstrate the effectiveness of DEGAS+SWiS in identifying high-risk regions within Xenium tissue samples (Fig. 1O).

## 6 Summary

This article demonstrated DEGAS’s seamless application across multiple scSRT platforms and diseases, including 10X Genomics Xenium and Nanostring’s CosMx and various disease types. Additionally, we implemented smoothing techniques, i.e. SWiS and FoVS, that account for spatial localization and created a novel pipeline for scSRT datasets. Initial experiments using a global k-nearest neighbors (kNN) approach for smoothing failed to capture finer spatial niche patterns. Therefore, we adopted the sliding-window approach, which allows the model to account for local microenvironmental effects by considering smaller neighborhoods. This approach preserves biologically meaningful local structures while mitigating noise in cell-level predictions. The application of DEGAS+SWiS/FoVS led to meaningful findings, such as identifying high-risk cell types in liver cancer (LIHC). Many genes associated with T2D were found to be significantly upregulated in high-risk cells identified by DEGAS+SWiS. These discoveries position DEGAS with spatial smoothing as a powerful and efficient tool for advancing translational research involving scSRT, offering significant potential for early diagnosis, prevention, and therapeutic interventions.

## Supplementary Material

btag098_Supplementary_Data

## Data Availability

The newly generated Type II Diabetes Xenium dataset underlying this article is available in Synapse under DOI: https://doi.org/10.7303/syn68699752. The Liver hepatocellular carcinoma CosMx dataset is publicly available from Bruker Spatial Biology at https://brukerspatialbiology.com/products/cosmx-spatial-molecular-imager/ffpe-dataset/human-liver-rna-ffpe-dataset/. The Melanoma Xenium dataset is publicly available from 10x Genomics at https://www.10xgenomics.com/datasets/human-skin-preview-data-xenium-human-skin-gene-expression-panel-1-standard.
