# Supplementary material for: Identification of high-risk cells in single-cell spatially resolved transcriptomics data using Diagnostic Evidence GAuge of Single-cells with spatial smoothing
Source: Bioinformatics. 2026 Apr 3;42(5):btag098. doi: 10.1093/bioinformatics/btag098 (PMC13184966; doi:10.1093/bioinformatics/btag098)
Supplement: btag098_Supplementary_Data [file btag098_supplementary_data.pdf]

Supplementary material for: Identification of High-Risk Cells in Single-Cell Spatially Resolved Transcriptomics Data Using DEGAS Spatial Smoothing

Debolina Chatterjee[1] Justin L. Couetil[2] Ziyu Liu[3] Kun Huang[2]
Chao Chen[4] Jie Zhang[2] Michael A. Kalwat[5] Travis S.Johnson[2]

[1] University of Mississippi Medical Center, Jackson, MS, USA
[2]Indiana University School Of Medicine, Indianapolis, IN, USA
[3]Purdue University, West Lafayette, IN, USA
[4]Stony Brook University, Stony Brook, NY, USA
[5] Indiana Biosciences Research Institute, Indianapolis, IN, USA

Abstract

This supplementary document contains a detailed description of some datasets, the relevant Algorithm, Tables, and Figures for the manuscript.

1 Supplementary Methods

1.1 Diagnostic Evidence Gauge of Single-cells (DEGAS) algorithm

Here we briefly describe the main steps in obtained disease association scores through the DEGAS pipeline (Johnson et al., 2022). The first step involves preprocessing of both the patient level bulk RNA-seq data, as well as the single-cell spatially resolved transcriptomic (scSRT) data. The set of highly variable genes was selected from the scSRT data, then the one that are common among those in the bulk RNA-seq data, is chosen. Next, the preprocessing of bulk RNA-seq and scSRT data involves filtering out cells that have low gene counts across the tissues, doing z-score normalization, min-max scaling, and log2 transformation of the counts to make the input matrices suitable for downstream analysis. This is done by the DEGAS function *preprocessCounts*, where a variance-stabilizing transformation was applied to reduce the influence of large count values while maintaining positive data. Each feature was then standardized using z-score normalization, where the mean was subtracted and the result divided by the standard deviation, with a small constant added to prevent division by zero. Following normalization, values were rescaled to the 0–1 range using min–max scaling to facilitate uniform feature scaling. The total loss consists of a maximum mean discrepancy (MMD)-based alignment loss used to match the latent distributions of bulk RNA-seq and scSRT data, together with a task-specific loss determined by the available annotations. Specifically, DEGAS uses a Cox proportional hazards loss when survival information is present, and a classification loss when patient-level or cell-level categorical labels are available. These loss combinations correspond to the LOSSClassCox, LOSSBlankCox, LOSSClassClass, LOSSClassBlank, and LOSSBlankClass formulations (Eqs. 7–11) described by Johnson et al. (2022). The inner architecture and mathematical details follow the original DEGAS framework.

1.2 Description of dataset used for the Type II Diabetes analysis

For the analysis in Section 3 of the main manuscript, de-identified formalin-fixed paraffin-embedded (FFPE) human pancreas tissue was obtained through the National Disease Research Interchange (NDRI) (Table 1). FFPE Tissue blocks were processed into 5 μm sections, and sections from two donors, two Non-diabetic (ND) and two type II Diabetic (T2D), were mounted directly within the bounding box of the Xenium slide by the Indiana University School of Medicine (IUSM) Histology Lab Service Core. Slides were processed according to the Xenium v1 workflow for in-situ Gene Expression (CG000582) using the Human Multi-tissue and Cancer panel (377 genes) by the IUSM Center for Medical Genomics (CMG) according to the instructions from 10X Genomics Xenium protocol (https://www.10xgenomics.com/). Data were processed and visualized in the Xenium Explorer software. The Xenium data for the four tissue samples can be found at DOI: https://doi.org/10.7303/syn68699752. Figures 1,2,3,4 show the volcano plots showing significantly upregulated (red) and downregulated (blue) genes considering Benjamini-Hochberg adjusted p-values ≤ 0.05 and threshold for the average log2 fold-changes in the expression of the genes appearing in at least 10% of cells in the Xenium samples. Tables 3, and 4 displays up- and down-regulated genes with their respective average log 2-fold changes in their expression values in the non diabetic (ND) and Type II diabetic (T2D) conditions respectively.

1.3 Description of the dataset used for Liver Hepatocellular Carcinoma (LIHC) analysis

This is the description of datasets for the analysis of Nanostring's CosMx data for Liver Hepatocellular Carcinoma (LIHC) samples in Section 4 of the main manuscript. The single-cell spatially re-

solved transcriptomics (scSRT) dataset was obtained from https://nanostring.com/products/cosmx-spatial-molecular-imager/ffpe-dataset/human-liver-rna-ffpe-dataset/, and is comprised of two FFPE tissue sections analyzed with the Human Universal Cell Characterization Panel (1000-plex). Sample 1 represents normal liver tissue (Male, Caucasian, Age 35), while Sample 2 corresponds to hepatocellular carcinoma tissue (Female, Caucasian, Age 65, Grade G3, Stage II). The gene expression metadata contains individual cell IDs, the field of view (FOV) pixel coordinates of the centroid of each cell, and the cell type of each individual cell. There are 22 unique cell types. The data contains 340,517 cells in the non-carcinoma sample and 464,126 cells in the LIHC sample. Figure 5 and 6 respectively show the various cell types which were identified in each tissue sample as provided in the publicly available data. The bulk RNA-seq data with clinical metadata were acquired from the TCGA LIHC study (https://www.cancer.gov/tcga), the files used for gene expression and clinical information are also presented in syn71737248. This bulk RNA-seq dataset included data from 377 human individuals along with their clinical information, including sex, age, overall survival status, and time.

1.3.1 Evaluation of DEGAS+smoothing

We applied copyKAT (Gao et al., 2021) to distinguish aneuploid from diploid cells within a subsample of 50,000 cells comprising the cell types $Hep.1, Hep.3, Hep.4, Hep.5$, and $Hep.6$ from the normal sample, and Hep, $tumor_1$, and $tumor_2$ from the carcinoma sample. copyKAT inferred chromosomal copy number variations to classify cells accordingly. However, the resulting annotations demonstrated low confidence, with aneuploid and diploid designations assigned to only 20,167 cells, and CNV scores obtained for 2,839 cells. See Figure 7 for the chromosome wise copy number alteration results results for the 50,000-cell subsample.

To assess the relationship between chromosomal instability and tumor association, we matched the copyKAT-inferred classifications with DEGAS-predicted LIHC association scores using the subsampled cell IDs. Among aneuploid cells, the mean LIHC association score was significantly higher than that of diploid cells (Wilcoxon test p = 0.0348, see Figure 8). Although copyKAT results exhibited low confidence due to limited gene coverage in the CosMx data set, the finding that DEGAS-predicted scores were significantly elevated in aneuploid cells supports the robustness of our approach in identifying high-risk cell populations within scSRT tissue samples. Figure 7 shows (A) the copyKAT classification results for the 50,000-cell subsample, and (B) a boxplot depicting the distribution of DEGAS-predicted LIHC association scores between aneuploid and diploid cells, along with the corresponding p-values.

Algorithm 1 DEGAS Impression Pipeline

Input: scSRT data, bulk RNA-seq data with clinical attributes
Output: Disease/subtype association scores (*impressions*)
procedure DEGAS PIPELINE

Step 1: Preprocessing:

- Align genes (intersection)
- Feature selection (e.g., least absolute shrinkage and selection operator (LASSO), t-tests)
- Normalize expression data (z-scores), scale to $[0, 1]$

Step 2: Model Selection:

- scSRT and bulk both have class labels **Use:** *ClassClass* model
- only bulk has class labels **Use:** *BlankClass* model
- only scSRT has class labels **Use:** *ClassBlank* model
- bulk has survival data **Use:** *BlankCox* model
- scSRT has class labels and bulk has survival data **Use:** *ClassCox* model

Step 3: Model Training:

- Build multitask deep model (e.g., DenseNet)
- Include losses: Cox (survival), classification (attributes), MMD (alignment)
- Perform bootstrap aggregation (BAg) m times
- Tune hyperparameters (batch sizes, dropout, regularization)

Step 4: Disease Attribute Mapping:

- Transfer patient-level attributes to single-cell data
- Generate disease association scores, i.e., impressions

end procedure

2 Supplementary Figures

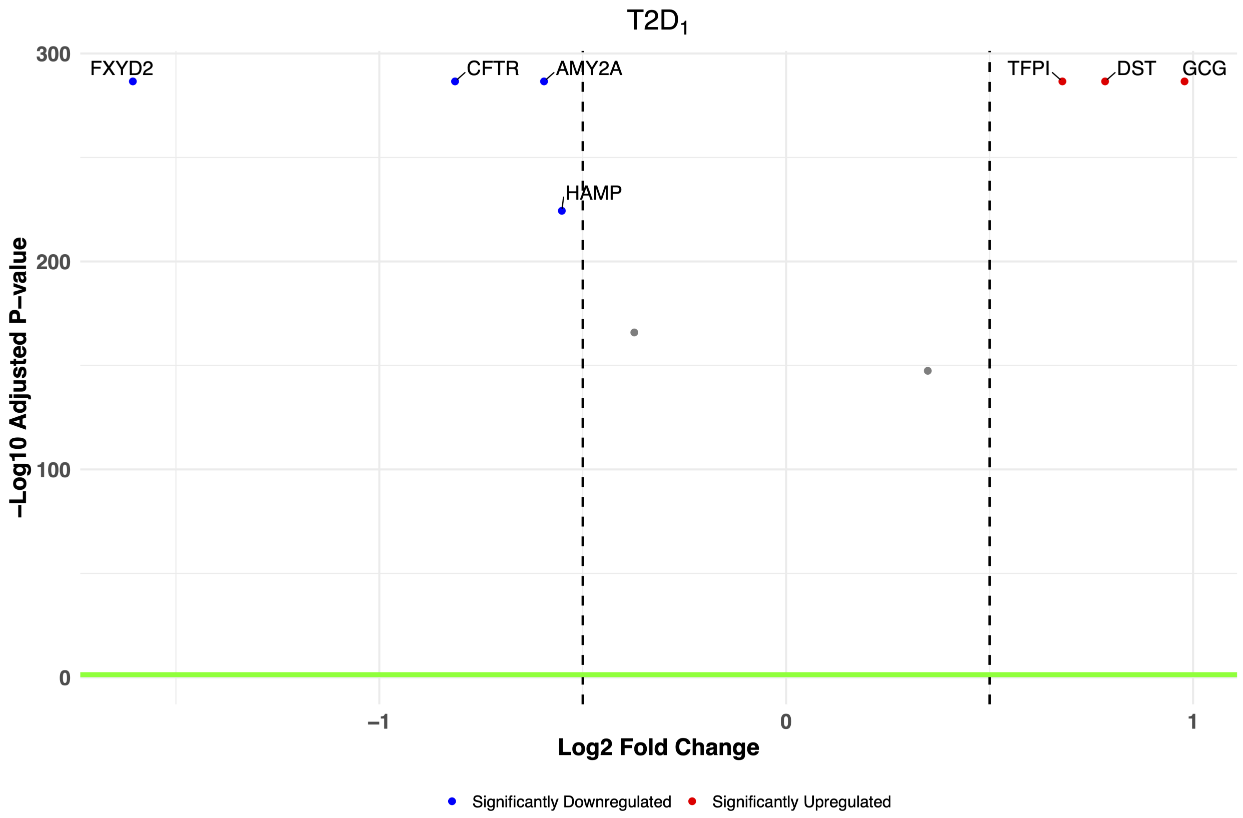

Figure 1: Volcano plots showing significantly upregulated (red) and downregulated (blue) genes considering Benjamini-Hochberg adjusted p-values ≤ 0.05 and threshold for the average log2 fold-changes in the expression of the genes appearing in at least 10% of cells in the Type II diabetic sample $T2D_1$.

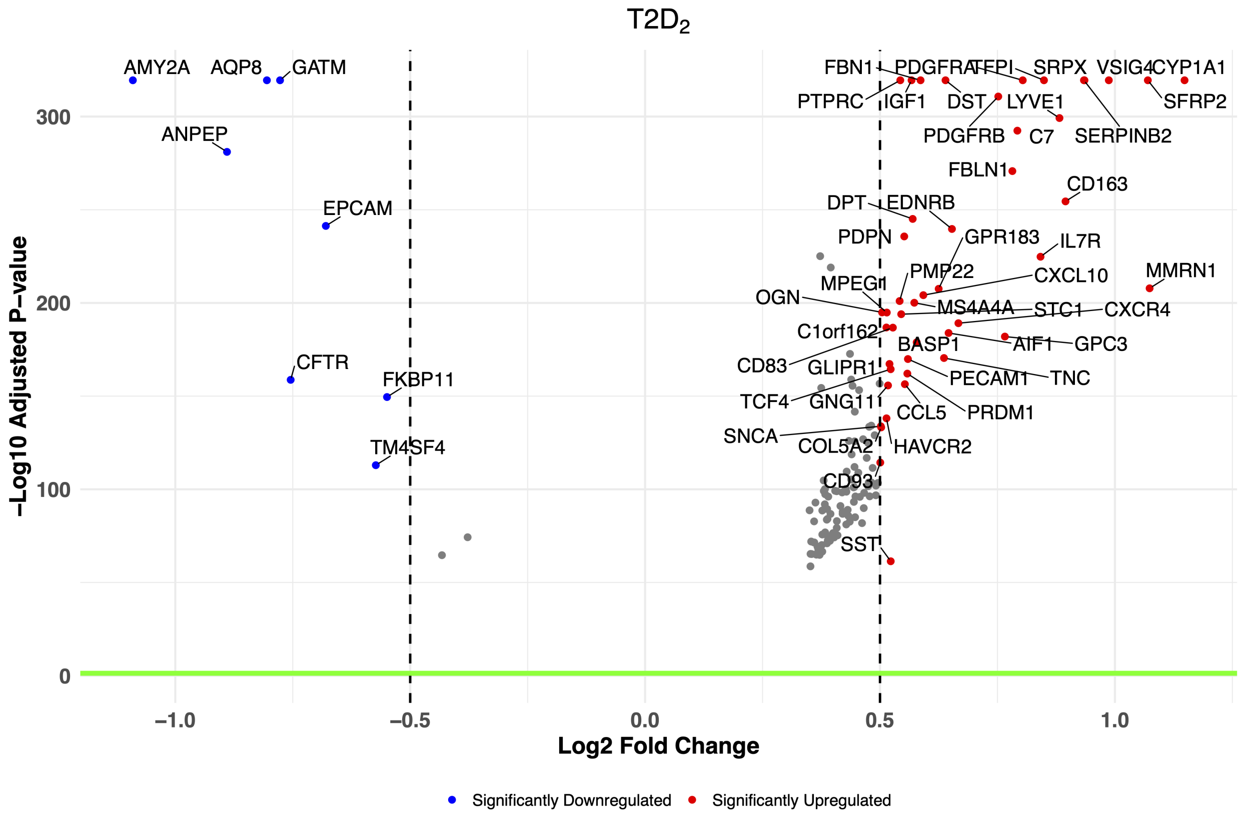

Figure 2: Volcano plots showing significantly upregulated (red) and downregulated (blue) genes considering Benjamini-Hochberg adjusted p-values ≤0.05 and threshold for the average log2 fold-changes in the expression of the genes appearing in at least 10% of cells in the Type II diabetic sample $T2D_2$.

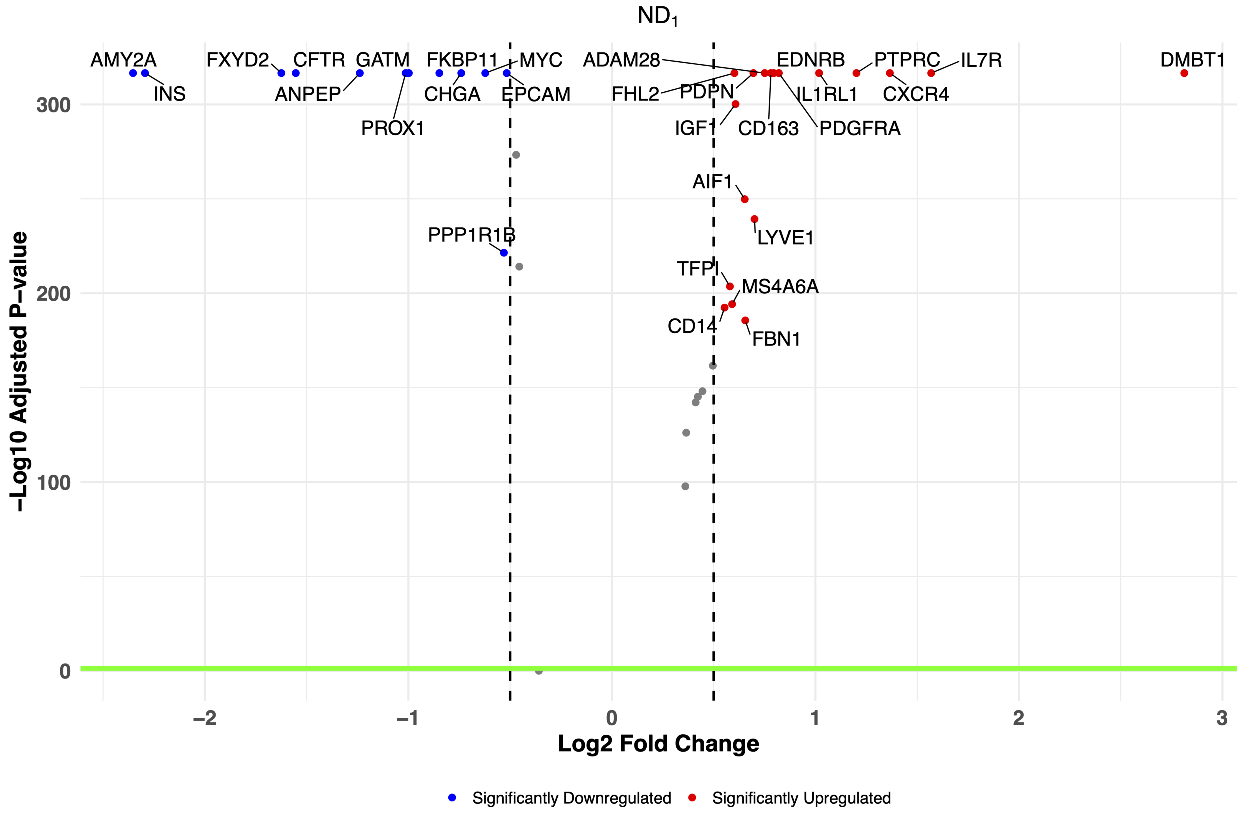

Figure 3: Volcano plots showing significantly upregulated (red) and downregulated (blue) genes considering Benjamini-Hochberg adjusted p-values ≤ 0.05 and threshold for the average log2 fold-changes in the expression of the genes appearing in at least 10% of cells in the Non-diabetic sample ND_1.

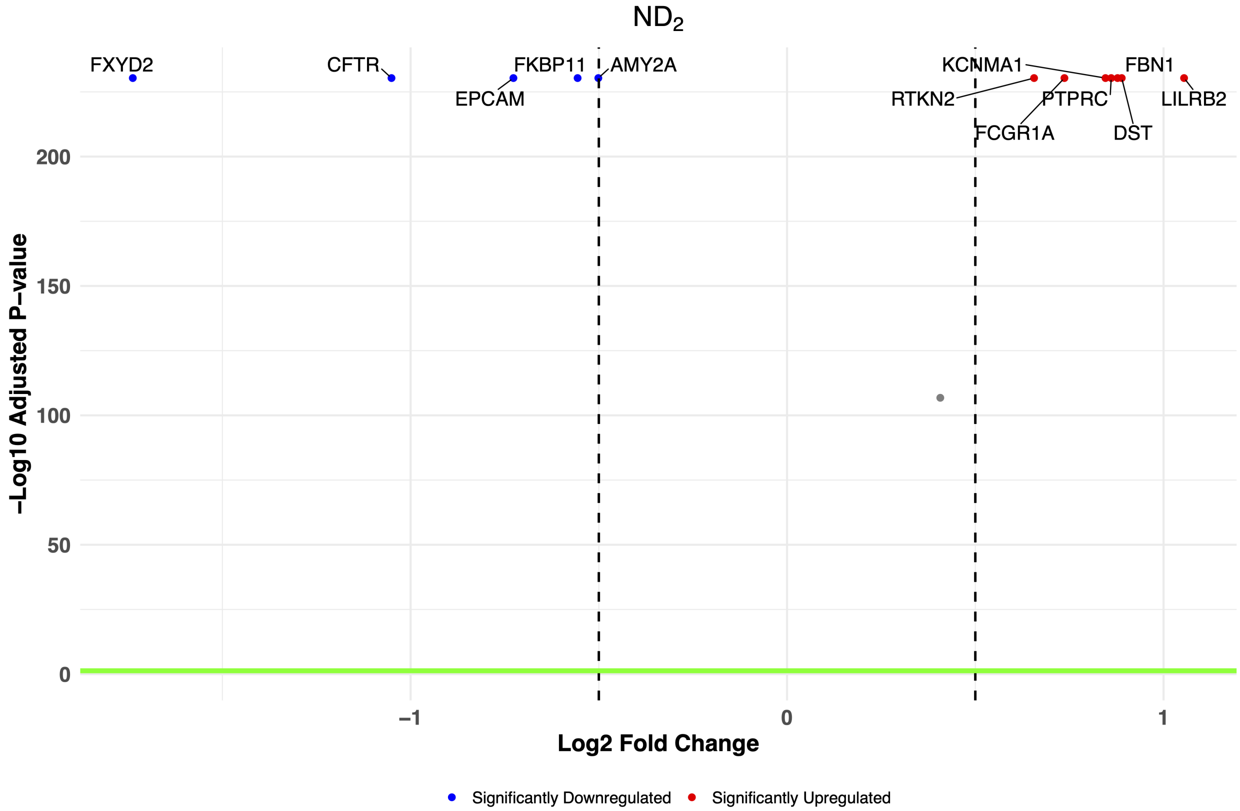

Figure 4: Volcano plots showing significantly upregulated (red) and downregulated (blue) genes considering Benjamini-Hochberg adjusted p-values ≤ 0.05 and threshold for the average log2 fold-changes in the expression of the genes appearing in at least 10% of cells in the Non-diabetic sample ND_2.

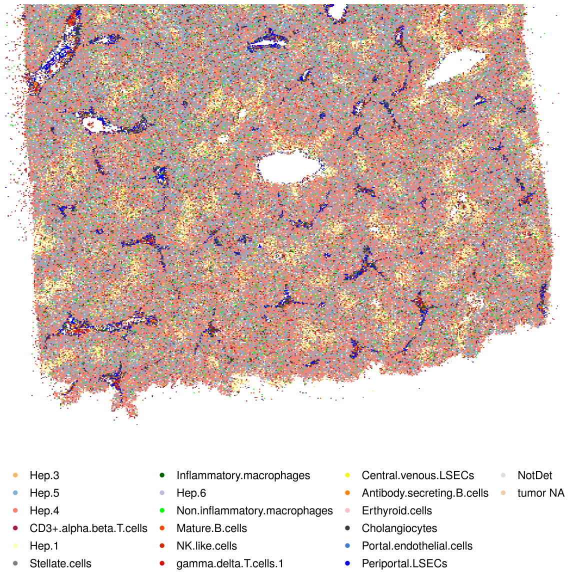

Figure 5: The Non-carcinoma sample showing various cell types. Source: https://nanostring.com/products/cosmx-spatial-molecular-imager/ffpe-dataset/human-liver-rna-ffpe-dataset/

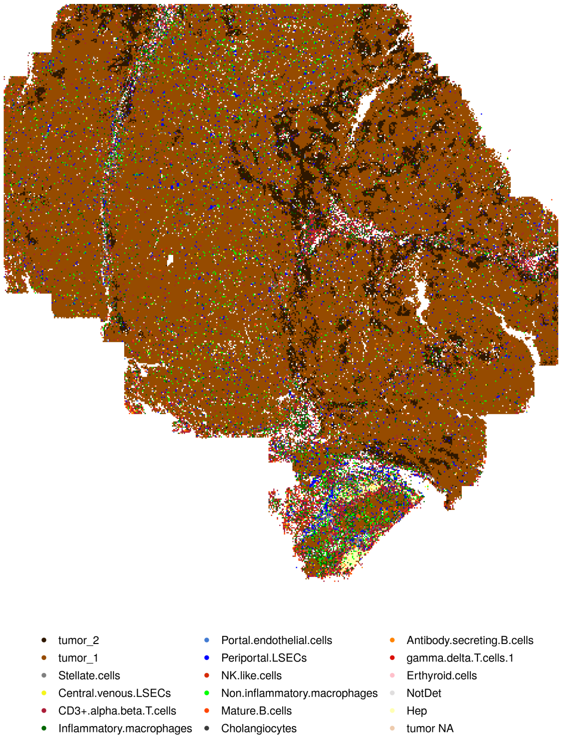

Figure 6: The Liver Hepatocellular Carcinoma (LIHC) sample showing various cell types. Source: https://nanostring.com/products/cosmx-spatial-molecular-imager/ffpe-dataset/human-liver-rna-ffpe-dataset/

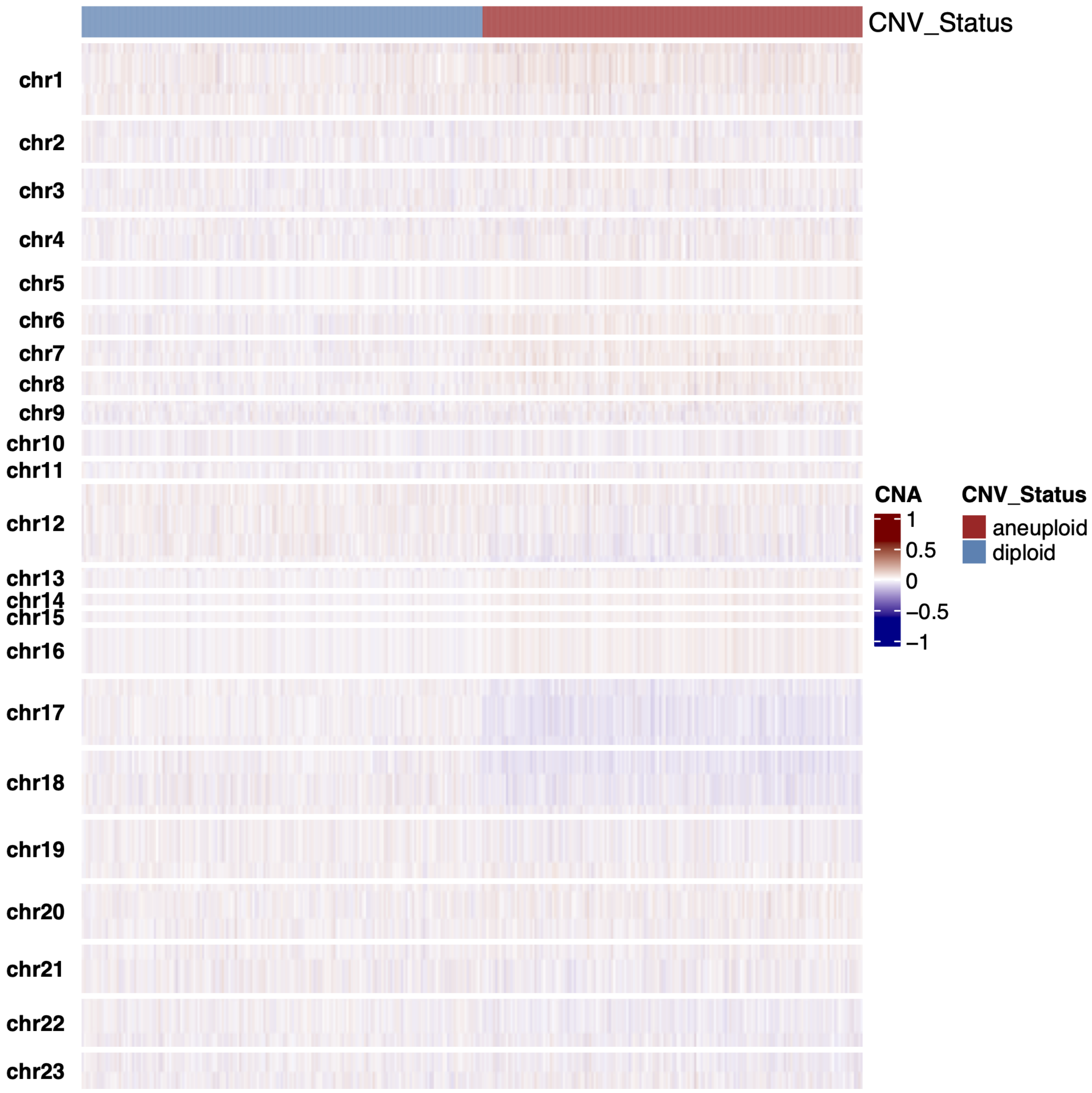

Figure 7: CopyKAT analysis of the 50,000-cell subsample from normal and LIHC carcinoma CosMx samples. Copy number alterations (CNAs) are shown across all chromosomes for cells classified as aneuploid (red) and diploid (blue). Red indicates regions of copy number gain, whereas blue denotes copy number loss.

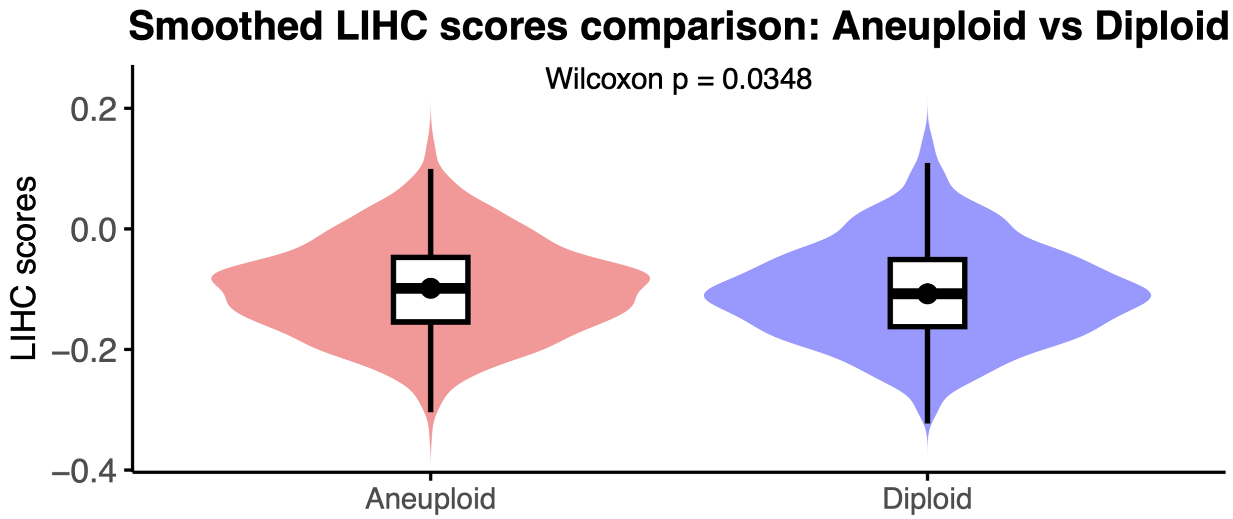

Figure 8: Boxplot showing the distribution of DEGAS + smoothing–derived LIHC association scores for cells classified as aneuploid and diploid by the copyKAT algorithm. The corresponding p-value from the Wilcoxon test is indicated.

3 Supplementary Tables

Table 1: Details of the Xenium Type II diabetes tissue samples. ICH stands for intracerebral hemorrhage

Sample ID	Gender	Race	Age	BMI	Diagnosis	Cause of Death	T2D Duration
ND_1	Male	White	82	28.4	Normal	Stroke, ICH	NA
$T2D_1$	Male	White	82	37.8	T2D	Respiratory arrest	30 yrs
$T2D_2$	Male	White	77	26.3	T2D	Stroke/ICH	15 yrs
ND_2	Female	White	78	23.7	Normal	Stroke, ICH	NA

Table 2: Empirical evaluation of multiple cutoff schemes to separate high- and low-risk cells. As compared to the other cutoffs (see table below), the 25th & 75th quantile cutoff provided the best trade-off between larger average of absolute value of the log2 fold changes (Log2FC) and a sufficient number of differentially expressed genes (DEGs).

Cutoff %	ND_1		ND_2		$T2D_1$		$T2D_2$	
	Avg $\|\log_2 \mathrm{FC}\|$	#DEGs	Avg $\|\log_2 \mathrm{FC}\|$	#DEGs	Avg $\|\log_2 \mathrm{FC}\|$	#DEGs	Avg $\|\log_2 \mathrm{FC}\|$	#DEGs
25th & 75th quantile	0.739	49	0.644	19	0.519	16	0.435	216
50th quantile	0.793	47	0.625	15	0.443	17	0.469	193
20th & 80th quantile	0.770	48	0.639	19	0.541	8	0.433	218

Table 3: Upregulated genes with their respective average log 2-fold changes in their expression values in the non diabetic (ND) and Type II diabetic (T2D) conditions.

**Upregulated genes**	ND_1	ND_2	$T2D_1$	$T2D_2$
DMBT1	2.813			
IL7R	1.569			0.842
PTPRC	1.202	0.861		0.543
EDNRB	0.795			0.653
IL1RL1	1.018			
CXCR4	1.366			0.667
PDPN	0.695			0.552
PDGFRA	0.82			0.804
CD163	0.781			0.895
ADAM28	0.751			
FHL2	0.602			
IGF1	0.607			0.567
AIF1	0.653			0.646
LYVE1	0.701			0.882
TFPI	0.58		0.679	0.849
MS4A6A	0.591			
CD14	0.554			
FBN1	0.655	0.878		0.586
DST		0.889	0.784	0.639
KCNMA1		0.889		
LILRB2		0.889		
FCGR1A		0.889		
RTKN2		0.656		
GCG			0.979	
CYP1A1				1.148
VSIG4				0.987
SRPX				0.935
SERPINB2				0.934
SFRP2				1.07
PDGFRB				0.752
C7				0.792
FBLN1				0.782
DPT				0.57

Continued on next page

Table 3 – continued from previous page

**Upregulated genes**	ND_1	ND_2	$T2D_1$	$T2D_2$
MMRN1				1.074
GPR183				0.625
CXCL10				0.592
PMP22				0.542
MS4A4A				0.573
OGN				0.504
MPEG1				0.514
STC1				0.545
C1orf162				0.514
CD83				0.527
GPC3				0.766
BASP1				0.578
TNC				0.636
PECAM1				0.559
GLIPR1				0.52
TCF4				0.523
PRDM1				0.558
CCL5				0.553
GNG11				0.517
HAVCR2				0.514
SNCA				0.502
COL5A2				0.502
CD93				0.501
SST				0.523

Table 4: Downregulated genes with their respective average log 2-fold changes in their expression values in the non diabetic (ND) and Type II diabetic (T2D) conditions.

**Downregulated genes**	ND_1	ND_2	$T2D_1$	$T2D_2$
AMY2A	-2.353	-0.501	-0.595	-1.09
CFTR	-1.553	-1.051	-0.814	-0.754
EPCAM	-0.517	-0.727		-0.68
INS	-2.294			
GATM	-0.998			-0.777
FXYD2	-1.624	-1.738	-1.606	
ANPEP	-1.239			-0.89
PROX1	-1.013			
FKBP11	-0.848	-0.556		-0.549
MYC	-0.622			
CHGA	-0.74			
PPP1R1B	-0.531			
HAMP			-0.551	
AQP8				-0.805
TM4SF4				-0.573

References

Gao, R., Bai, S., Henderson, Y. C., Lin, Y., Schalck, A., Yan, Y., Kumar, T., Hu, M., Sei, E., Davis, A., et al. (2021). Delineating copy number and clonal substructure in human tumors from single-cell transcriptomes. *Nature biotechnology*, 39(5):599–608.

Johnson, T. S., Yu, C. Y., Huang, Z., Xu, S., Wang, T., Dong, C., Shao, W., Zaid, M. A., Huang, X., Wang, Y., et al. (2022). Diagnostic evidence gauge of single cells (degas): a flexible deep transfer learning framework for prioritizing cells in relation to disease. *Genome medicine*, 14(1):11.
